# Prognostic impact of Claudin 18.2 in gastric and esophageal adenocarcinomas

**DOI:** 10.1007/s12094-020-02380-0

**Published:** 2020-06-01

**Authors:** A. Arnold, S. Daum, M. von Winterfeld, E. Berg, M. Hummel, B. Rau, U. Stein, C. Treese

**Affiliations:** 1 Institute of Pathology, Charité-Universitätsmedizin Berlin, Berlin, Germany; 2 Department of Gastroenterology, Infectious Diseases and Rheumatology. Campus Benjamin Franklin, Charité-Universitätsmedizin Berlin, Hindenburgdamm 30, 12203 Berlin, Germany; 3 Berlin Institute of Health (BIH), Berlin, Germany; 4 Institute of Pathology Heidelberg, University Hospital Heidelberg, Heidelberg, Germany; 5 Department of Surgery. Campus Virchow-Klinikum and Campus Mitte, Charité-Universitätsmedizin Berlin, Berlin, Germany; 6 Experimental and Clinical Research Center, Charité-Universitätsmedizin Berlin and Max-Delbrück-Center for Molecular Medicine in the Helmholtz Association, Berlin, Germany; 7 German Cancer Consortium (DKTK), Heidelberg, Germany

**Keywords:** Claudin 18.2, Claudiximab, IMAB362, Gastric cancer, Esophageal cancer

## Abstract

**Introduction:**

The tight junction molecule Claudin 18.2 is selectively expressed in healthy and malignant gastric epithelial tissue and is a promising therapy target for high Claudin 18.2 expressing adenocarcinomas of the esophagogastric junction and stomach (AEG/S).

**Methods:**

This study analyzed the prevalence, characteristics and prognostic impact of Claudin 18.2 expression in primary tumor, lymph node and distant metastasis in a large Caucasian AGE/S cohort with 414 patients.

**Results:**

Claudin 18.2 was highly expressed in 17.1% of primary tumors, 26.7% of lymph node metastasis and 16.7% of distant metastasis. High Claudin 18.2 expression in lymph node metastasis and primary tumors correlated significantly (*p* < 0.001). High expression of Claudin 18.2 was neither associated with histomorphogical subtype, or tumor state, nor with overall survival.

**Conclusion:**

In Caucasian AEG/S patients, 17.1% appeared to be eligible for an anti-Claudin 18.2 therapy. Claudin 18.2 expression itself has no impact on prognosis and is not related to any tumor subtype.

**Electronic supplementary material:**

The online version of this article (10.1007/s12094-020-02380-0) contains supplementary material, which is available to authorized users.

## Introduction

In 2018, about 783,000 people died due to adenocarcinoma of the esophagogastric junction and stomach (AEG/S) worldwide [1]. Despite an increasing number of targeted-therapy options in many tumor entities, the therapeutic options in AEG/S are limited to cytotoxic chemotherapy, anti-Her2- and anti-VEGFR2 strategies [2, 3].

Sahin et al. (2008) identified the tight junction molecule Claudin-18 isoform 2 as a promising target in AEG/S therapy [4]. They found that the isoform Claudin 18.2 is strictly expressed in differentiated epithelial cells of the gastric mucosa and also in 75% of AEG/S.

In phase I and IIa clinical trials, the therapeutic use of the monoclonal anti-Claudin 18.2 antibody Claudiximab (IMAB362) was well tolerated and the therapy demonstrated a 10% response rate, a 30% disease control rate in a monotherapy PHASE II study (MONO trial, NCT01197885), and a response rate of 39% in a combination PHASE II study with epirubicin, oxaliplatin, and capecitabine (EOX) (FAST trial, NCT01630083) [5, 6]. Due to the promising phase I/II data, several phase III studies are underway (NCT03528629, NCT03505320, NCT03653507, NCT03504397 (SPOTLIGHT)).

The characteristic of Claudin 18.2-positive AEG/S tumors have been recently analyzed in three retrospective studies. The first Japanese study detected a medium to high Claudin 18.2 expression in 51.5% of their patients. Claudin 18.2 expression was correlated with a diffuse histologic subtype [7]. Another study conducted by Dottermuch et al. was performed in a large Caucasian gastric cancer cohort of 481 patients [8]. In contrast to the MONO trial and to the analysis of Rhode et al., they used as CLAUDIN 18.2 antibody the clone EPR19202. In this study, they could detect a significantly increased expression (> 50% positive tumor cells, intensity 2 +) of Claudin 18.2 in only two patients (0.4%). The most recent study from Caoti et al. using clone 34H14L15 and including a cohort of 523 AEG/S patients detected high Claudin expression in 29.4% of patients. Moreover, in their study Claudin 18.2. expression was correlated with a diffuse histologic subtype, corpus localization and EBV-associated subtype [9].

In summary, Claudin 18.2 is a tight junction molecule selectively expressed in gastric epithelial cells and seems to be a promising target in AEG/S. Although Claudin 18.2 has been thoroughly characterized, solid survival data that are crucial for the analysis of the prognostic impact of Claudin 18.2 are still missing.

This study analyzes the prognostic impact of Claudin 18.2 expression in a large retrospective AEG/S cohort with a long follow-up time. Furthermore, we compared both antibodies, clone 43-14A applied in the FAST trial and in the ongoing SPOTLIGHT trial as well as the clone EPR19202, used by Dottermusch et al., to understand the differences in expression frequency of the previous studies.

## Materials and methods

### Patients

Clinical data from 414 patients with AEG/s of all tumor stages, primarily treated by surgery between 1992 and 2004 at the Charité—Universitätsmedizin Berlin, were collected retrospectively. The mean follow-up was 121.7 months (95% CI 113.9–129.5). The data including patient characteristics and follow-up information were retrieved from the patient management software (SAP®) and the regional population-based cancer registry (“Gemeinsames Krebsregister”) and are summarized in Table 1. This study was approved by the Institutional Review Board of the Charité (EA4/115/10).

**Table 1:** Patient characteristics of the analyzed patient cohort and distribution of Claudin 18.2-positive and -negative primary tumors

	All		Claudin 18.2				*p*
	Total		Neg		Pos		
	*n*	(%)	*n*	(%)	*N*	(%)	
Gender							
Female	157	(41.2)	136	86.6	21	13.4	0.071
Male	224	(58.8)	180	80.4	44	19.6	
Age group							
< 65 years	215	(56.4)	179	83.3	36	16.7	0.891
> = 65 years	166	(43.6)	137	82.5	29	17.5	
Localization							
Gastric Cancer	325	(85.3)	267	82.2	58	17.8	0.218
AEG	56	(14.7)	49	87.5	7	12.5	
Tumor stage							
T1	91	(23.9)	73	80.2	18	19.8	0.147
T2	152	(39.9)	130	85.5	22	14.5	
T3	106	(27.8)	89	84.0	17	16.0	
T4	31	(8.1)	24	77.4	7	22.6	
Unknown	1	(0.3)	0	0.0	1	100.0	
Node stage							
N0	158	(41.5)	128	81.0	30	19.0	0.400
N +	223	(58.5)	188	84.3	35	15.7	
Distant metastasis							
M0	288	(75.6)	235	81.6	53	18.4	0.472
M1	85	(22.3)	74	87.1	11	12.9	
Unknown	8	(2.1)	7	87.5	1	12.5	
Lymphatic vessel invasion							
L0	138	(36.2)	111	80.4	27	19.6	0.103
L1	188	(49.3)	161	85.6	27	14.4	
Unknown	55	(14.4)	–		–		
Vein invasion							
V0	214	(56.2)	179	83.6	35	16.4	0.169
V1	105	(27.6)	88	83.8	17	16.2	
Unknown	62	(16.3)	–		–		
Grading							
G1	8	(2.1)	7	87.5	1	12.5	0.661
G2	105	(27.6)	84	80.0	21	20.0	
G3	265	(69.6)	223	84.2	42	15.8	
Unknown	3	(0.8)					
Lauren classification							
Intestinal	160	(42.0)	131	81.9	29	18.1	0.696
Diffuse	167	(43.8)	142	85.0	25	15.0	
Mixed	51	(13.4)	41	80.4	10	19.6	
Unknown	3	(0.8)					
Ming classification							
Expansive	158	(41.5)	131	82.9	27	17.1	0.182
Infiltrative	216	(56.7)	181	83.8	35	16.2	
Unknown	7	(1.8)					
Her2Neu							
Neg	303	(79.5)	248	81.8	55	18.2	0.501
Pos	29	(7.6)	26	89.7	3	10.3	
Unknown	49	(12.9)	–	–	–	–	
MMR							
Proficient	316	(79.5)	198	62.7	118	37.3	0.310
Deficient	38	(7.6)	27	71.1	11	28.9	
Unknown	27	(12.9)	–	–	–	–	

Significance calculated by *X*^2^ test

### Tissue samples

Out of FFPE tumor samples from 414 patients (primary tumors *n* = 392, synchronous lymph node metastasis *n* = 151 and synchronous distant metastasis *n* = 40), tissue-micro arrays (TMA) were engineered and analyzed histomorphologically as described before [10]. Immunohistochemical analysis was performed on TMA sections using two different Claudin 18.2-specific monoclonal antibodies: clone EPR19202 (Abcam, Cambridge, UK, dilution: 1:500) and clone 43-14A (Roche Ventana Medical Systems, dilution: 1:1). The immunostaining was carried out using the Leica Bond-Max Autostainer (Leica Biosystems. IL, USA) according to the manufacturer’s protocol. After heat-induced epitope retrieval, the sections were incubated with the described antibodies. Horseradish peroxidase-labeled anti-rabbit-IgG using the Bond Polymer Detection Kit (Leica Biosystems. IL, USA) was employed to uncover the chromogen substrate.

Expression was evaluated by an immunoreactivity score (IRS): percentage of stained tumor cells (0 = 0%. 1 = 1–25%. 2 = 26–50%. 3 = 51–75%. 4 = 76–100%) was multiplied with the staining intensity (score 0–3 = no staining to strong staining) to give the IRS score of each sample (score 0–12). Samples with IRS > 8 were assessed as Claudin 18.2-positive tumors, and samples with < / = 8 as Claudin 18.2-negative tumors.

HER2 expression was determined by immunohistochemistry using a monoclonal anti-HER2 antibody (clone 4B5; Ventana Medical Systems). HER2 status was determined according to the consensus panel recommendation on HER2 testing in gastric cancer [11].

### Statistics

Statistical analysis was performed using IBM SPSS Version 24. Overall survival was defined as time from diagnosis to death or last follow-up and was compared using Kaplan–Meier method with the log-rank test for assessment of statistical significance.

Associations of Claudin 18.2 expression with tumor size, distant and lymph node metastasis, venous and lymphatic infiltration, Lauren and Ming classification, grading and UICC classification were tested using the *χ*^2^ test.

## Results

### Clinical characteristics

The detailed clinicopathological characteristics are summarized in Table 1.

### Claudin 18.2 expression in primary tumors using clone EPR19202

Using clone EPR19202, Claudin 18.2 staining was evaluable in 381 of 392 primary tumors (97.2%), 146 of 151 lymph node metastases (96.7%) and 36 of 40 distant metastases (90.0%). Staining with clone EPR19202 resulted in a weak staining. No sample reached an IRS > 8. Eight samples (2.1%) were scored with an IRS 4–6 and 15 (3.9%) with IRS 1–3 (see Figure S1).

### Claudin 18.2 expression in primary tumors using clone 43-14A

**Fig. 1:**
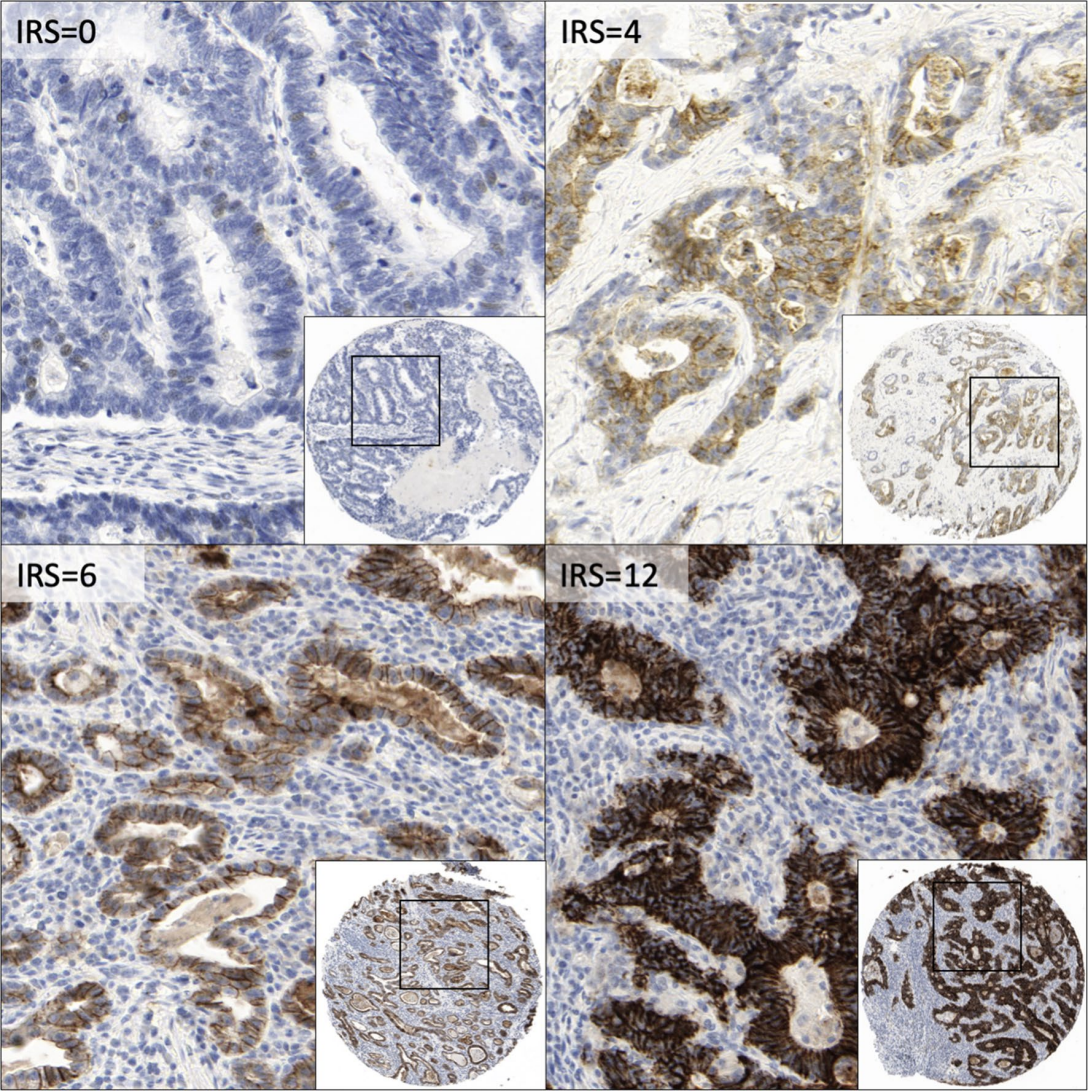
Representative Claudin 18.2 IHC staining of TMA cores using the anti-CLDN 18.2 clone 43-14A. Examples of tumor samples with IRS = 0.4.6 and 12 (100 × and 400 × magnitude)

From 392 primary tumor samples, 381 samples (97.2%) were evaluable after staining with Clone 43-14A. High Claudin 18.2-positive tumor samples, defined by an IRS > 8, were identified in 65 cases (17.1%) (Figure 1). Samples were scored in 47.0% (*n* = 179) as IRS = 0, in 14.2% (*n* = 54) as IRS 1–3, in 21.8% (*n* = 83) as IRS 4–8 and in 17.1% (*n* = 65) IRS > 8. There was no difference in Claudin 18.2 expression between old and younger FFPE samples (× 2 *p* = 0.581).

**Fig. 2:**
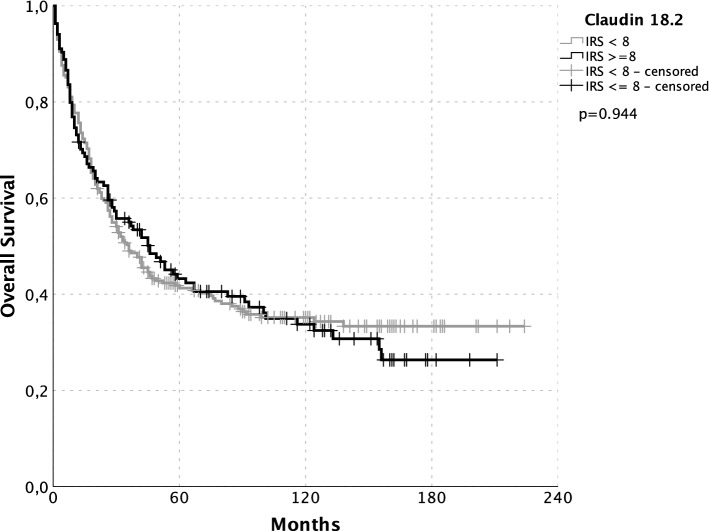
Kaplan–Meier plots of overall survival. Claudin 18.2 neg.: gray; pos.: black. Significance calculated by log rank: no significant differences in survival between Claudin 18.2-negative (black) and -positive (gray) patients (*p* = 0.944)

The comparison of both antibodies showed that high scored samples in the 43-14A staining were those samples which also showed the highest IRS with use of EPR19202 (see Table S1) (× 2 *p* < 0.0001). The correlation of Claudin 18.2 expression status and patient characteristics was negative (see Table 1). There were no differences in overall survival or disease-specific survival between Claudin 18.2-positive and -negative patients (see Fig. 2).

### Claudin 18.2 expression in lymph node and distant metastasis

From 151 lymph node samples, staining was evaluable in 149 samples (98.7%). Claudin 18.2 expression was identified in 39 of 146 lymph node metastasis samples (26.7%). 116 of these samples could be matched with the primary tumor samples from the same patient. In 78.1% (*n* = 68), Claudin 18.2-negative lymph node metastasis had corresponding negative primary tumor and in 70.1% (*n* = 23) Claudin 18.2-positive lymph node metastasis corresponded with positive primary tumors (*p* < 0.0001) (see Table 2).

**Table 2:** Distribution of Claudin 18.2-negative and -positive stained primary tumors and their correspondent lymph node (N) and distant metastasis (M)

	**CLDN 18.2**	Negative	Positive	*p *value (× 2)
N	Negative	68 (78.1%)	19 (21.8%)	
	Positive	6 (20.7%)	23 (70.1%)	< 0.0001
M	Negative	11 (68.8%)	5 (31.2%)	
	Positive	1 (100%)	0 (0.0%)	0.506

Significance calculated by *X*^2^ test

From 40 distant metastasis samples, staining was evaluable in 36 samples (90.0%). Claudin 18.2 expression was identified in 6 of 36 distant metastasis samples (16.7%). Twelve of the 63 distant metastasis samples had corresponding primary tumor samples from the same patient. In 68.8% (*n* = 11), Claudin 18.2-negative distant metastasis had the corresponding negative primary tumor and in 0% (*n* = 0) Claudin 18.2-positive distant metastasis had correspondent positive primary tumors (*p* = 0.506) (see Table 2).

## Discussion

Tight junction molecule Claudin 18.2 has been found to be a promising target in AEG/S therapy as it is only expressed in healthy and some cases of malignant gastric epithelial tissue [4].

The present study analyzed the frequency of high Claudin 18.2 expression, the prognostic impact and the correlation with histo-morphological risk groups in a large Caucasian AEG/S population. Using clone 43-14A for immunostaining, we detected 17.1% patients with a high Claudin 18.2 expression, which is similar to the results of the MONO trial (14.4%) [6]. These data are not congruent with data from a Japanese Gastric cancer cohort which detected in 135 of 262 cases a strong Claudin 18.2 expression (51.5%) [7]. The differences of expression between our Caucasian cohort and the Japanese cohort might be an effect of ethnical difference in Claudin 18.2 expression and will be elucidated by the data of the international recruiting NCT03504397 Trial (Spotlight).

The differences between our data and the data from Dottermusch et al. seem to be related to the use of the different antibodies used for IHC. When we used the same clone as Dottermusch et al. [8] (EPR19202), we got the same weak staining intensity as descripted by the authors. Table S1 makes the differences of EPR19202 and 43-14A clear and shows that the sensitivity of 43-14A is higher and should be used for Claudin 18.2 diagnostics. The results from Claudin 18.2 expression analysis in lymph node and distant metastasis indicate that Claudin 18.2 diagnostics should be performed on primary tumors and lymph node metastasis, but not on distant metastasis.

Our data show that the expression of Claudin 18.2 in Caucasian AEG/S patients is not associated with overall survival and is not related to any histo-morphological subtype. In summary, Claudin 18.2 is not a prognostic biomarker regarding the REMARK criteria [12]. Outside of a potential claudiximab therapy, the expression of Claudin 18.2 does not contain any information that is useful for disease management. This result is consistent with the fact that Claudin 18.2 is not part of any cancer-related pathway and the effect of anti-Claudin 18.2 therapy is raised by antibody-dependent cellular cytotoxicity [13]. Clinical trials must show whether the expression of Claudin 18.2 is predictive for therapy with claudiximab.

## Code availability

Not applicable.

## Electronic supplementary material

Below is the link to the electronic supplementary material.

Supplementary file1 (DOCX 13 kb)

Supplementary file2 (TIFF 5746 kb)

## Data Availability

Data are available as SPSS file in the electronic supplement.
