# Supplementary material for: Prognostic impact of Claudin 18.2 in gastric and esophageal adenocarcinomas
Source: Clin Transl Oncol. 2020 Jun 1;22(12):2357–63. doi: 10.1007/s12094-020-02380-0 (PMC7577914; doi:10.1007/s12094-020-02380-0)
Supplement: Supplementary file 1 — Supplementary file1 (DOCX 13 kb) [file 12094_2020_2380_MOESM1_ESM.docx]

|  |  | clone 43-14A | | | |  |
| --- | --- | --- | --- | --- | --- | --- |
| clone EPR19202 | IRS | 0 | 1-3 | 4-8 | 9-12 | all |
|  | 0 | 176 | 54 | 77 | 51 | 358 |
|  | 1-3 | 3 | 0 | 6 | 6 | 15 |
|  | 4-8 | 0 | 0 | 0 | 8 | 8 |
|  | 9-12 | 0 | 0 | 0 | 0 | 0 |
|  | all | 179 | 54 | 83 | 65 | 381 |

Table S1: Comparison of Staining evaluation using clone EPR19202 and clone 43-14A.
