# Supplementary figures and images for: Prognostic impact of Claudin 18.2 in gastric and esophageal adenocarcinomas
Source: Clin Transl Oncol. 2020 Jun 1;22(12):2357–63. doi: 10.1007/s12094-020-02380-0 (PMC7577914; doi:10.1007/s12094-020-02380-0)

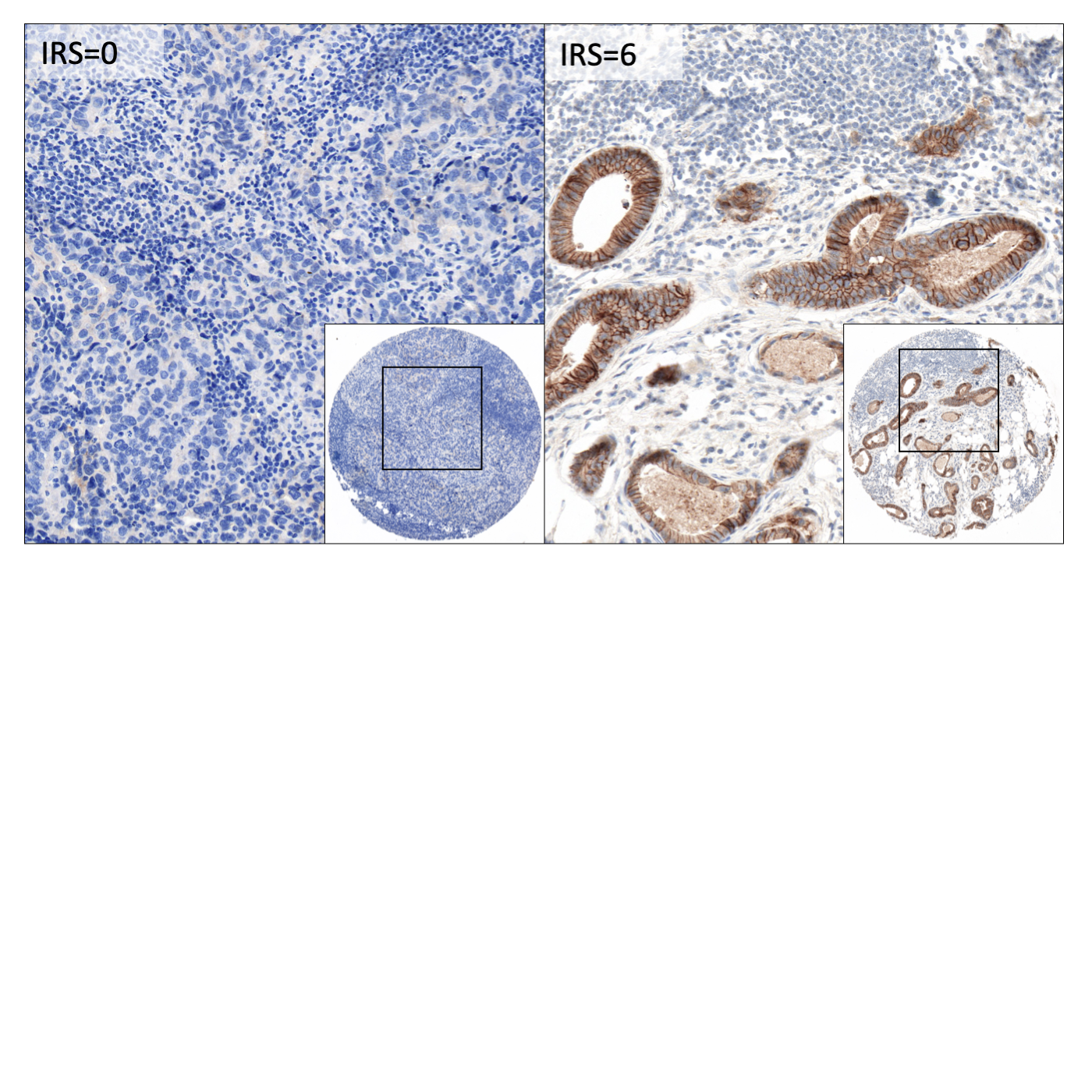

Supplement: Supplementary file 2 — Supplementary file2 (TIFF 5746 kb) [file 12094_2020_2380_MOESM2_ESM.tiff]
